# Manifestations neuropsychiatriques révélant une hémorragie cérébro-méningée causée par un accident d’électrisation: à propos d'une observation et revue de la literature

**DOI:** 10.11604/pamj.2014.18.201.4832

**Published:** 2014-07-05

**Authors:** Marcellin Bugeme, Olivier Mukuku

**Affiliations:** 1 Cliniques Universitaires de Lubumbashi, Faculté de Médecine, Université de Lubumbashi, RDCongo; 2 Centre Neuropsychiatrique Dr Joseph Guislain/Frères de la Charité, Lubumbashi, RD Congo

**Keywords:** Electrisation, hémorragie cérébro-méningée, œdème cérébral, electrocution, cerebro-meningeal haemorrhage, cerebral hematoma

## Abstract

Le courant électrique est susceptible de léser tout tissu de l'organisme rencontré lors de son passage, de manière transitoire ou définitive. Les hémorragies cérébro-méningées secondaires à un accident d’électrisation par courant électrique à haute tension sont très rarement rapportées dans la littérature. Nous rapportons un cas d'hémorragie cérébro-méningée révélée par des manifestations neuropsychiatriques causée par un AE par courant électrique à haute tension observée chez un enfant âgé de 6 ans à Lubumbashi, en République Démocratique du Congo. La particularité que présente notre observation est les manifestations neuropsychiatriques observées tardivement.

## Introduction

Encore appelé électro-traumatisme, l’électrisation correspond à toutes les manifestations physiologiques et physiopathologiques dues au passage du courant électrique au travers du corps humain [1–3]. Les données épidémiologiques relatives aux accidents d’électrisation (AE) sous-estiment probablement leur incidence réelle car beaucoup d’événements mineurs survenant au travail ou dans le cadre domestique ne donnent pas lieu à une consultation médicale ou à une déclaration d'accident [4]. En Suisse, environ 110 AE professionnels sont annoncés chaque année et aux Etats-Unis, on l'estime à 70 cas pour 100 000 habitants/an [5]. Sur le parenchyme cérébral, les effets de l’électricité entrainent des manifestations neurologiques nombreuses qui peuvent être soit centrales ou périphériques, d'apparition immédiate ou retardée et transitoires ou définitives [6]. Deux principaux types d′AE en fonction de la tension du courant électrique sont à distinguer: (a) AE par courant à basse tension lorsque le voltage est en dessous de 1000 volts (essentiellement des accidents domestiques, dont les deux tiers des victimes sont des enfants) et (b) AE par courant à haute tension lorsqu'il est au-dessus de 1000 volts (survenant surtout lors d′accidents du travail et l′homme jeune est le plus souvent concerné par ce type de traumatisme) [7]. La mortalité due aux AE varie entre 2-3% pour les AE à basse tension et entre 5-30% pour les AE à haute tension [8]. Les hémorragies cérébro-méningées secondaires à un AE par courant électrique à haute tension sont très rarement rapportées dans la littérature et à notre connaissance une seule observation a été publiée jusqu’à présent [8]. Nous rapportons un cas d'hémorragie cérébro-méningée révélée par des manifestations neuropsychiatriques causée par un AE par courant électrique à haute tension observée chez un enfant âgé de 6 ans à Lubumbashi, en République Démocratique du Congo. La particularité que présente notre observation est les manifestations neuropsychiatriques observées tardivement.

## Patient et observation

O.K., âgé de 6 ans, élève en première année primaire dont la mère est venue consulté le Centre Neuropsychiatrique Dr Joseph-Guislain pour un trouble de comportement fait d'agressivité avec passage à l'acte, d'agitation, de dysharmonie dans son comportement alimentaire, nervosité et refus d'obtempérer aux ordres des parents, de déambulation avec refus catégorique de retourner à son domicile. Le début de l'histoire de la maladie remontait à deux mois de ces manifestations, quand l'enfant revenait de l’école avec ses amis et se dirigea par curiosité dans une cabine de transformation du courant électrique de haute tension en basse tension qui était implantée sur son passage; une fois dans cette cabine, l'enfant sera électrisé et projeté en dehors cette dernière. Ce sont les passagers qui le prendront dans un état d'inconscience qui avait duré quelques 5 à 10 minutes et l'acheminera dans une structure médicale où les premiers soins de réanimation ont été administrés. Ce n'est qu'après 24 heures que le patient va se rétablir, commença à marcher et à parler, ainsi il expliqua lui-même les faits et dira qu'il avait senti une sorte d'aimantation entre lui et le fil électrique qu'il avait touché, puis un relâchement brusque (surement que c’était une contraction musculaire qui « gèle » la victime sur la source de contact (c'est le « can not let go » des anglo-saxons). Deux mois plus tard, l'enfant manifestera un trouble de comportement et ceci va motiver la mère de l'enfant à consulter le Centre Neuropsychiatrique Dr Joseph-Guislain pour une prise en charge. Ses antécédents ne montrent rien de particulier.

L'anamnèse révélait que l'enfant refuse de se laver et de manger par moment sans raison aucune et présente un rire immotivé. L'examen neuropsychiatrique mettait en évidence une altération de fonctions intellectuelles et un état confusionnel (une altération de la mémoire d’évocation et de fixation, une désorientation temporo-spatiale), une marche ébrieuse, une agitation et instabilité (il faisait le 100 pas), un refus catégorique d'obéir aux ordres. Sur le plan somatique, nous avons objectivé des cicatrices de brûlures cutanées, à prédominance deuxième degré, ayant intéressé environ 15% de la surface corporelle, siégeant au niveau du 1/3 antéro-externe de l'avant-bras droit, au niveau du gril costal droit (entre la 2ème et la 7ème côte) et au niveau des orteils de deux côtés. Aucune lésion cutanée objectivée au niveau du crâne. Nous avions conclu à un trouble psychotique avec excitation psychomotrice associé à un syndrome cérébelleux post AE et nous avons instauré un traitement fait de Pipamperone (15 gouttes deux fois par jour), Chlorpromazine (25 mg deux fois par jour).

**Figure 1:**
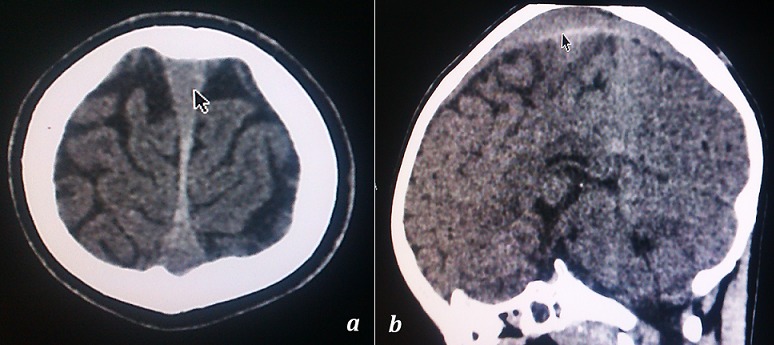
(a) et (b) Hémorragie sous-durale essentiellement dans la faux du cerveau. Pas de lésion osseuse

**Figure 2:**
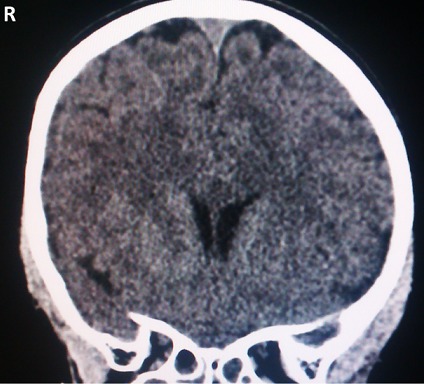
Hypodensité de la substance blanche avec ventricule latéral droit d'aspect comprimé comparé au gauche suggérant un oedème cerebral

Le scanner cérébral réalisé n'avait pas mis en évidence de lésion osseuse et avait révélé une hémorragie sous-durale essentiellement dans la faux du cerveau (8 mm épaisseur), une nette hypodensité de la substance blanche avec ventricule latéral droit d'aspect comprimé comparé au gauche suggérant un oedème cérébral, sans déviation de la ligne médiane et des sillons pariétaux droits moins exprimés (Figure 1 et Figure 2). L’électroencéphalogramme réalisé était perturbé par l'abondance en éléments lents diffus. L’électrocardiogramme était normal. Au vu de tous ces éléments, nous avions conclu à une hémorragie sous-durale et un oedème cérébral post AE.

**Figure 3:**
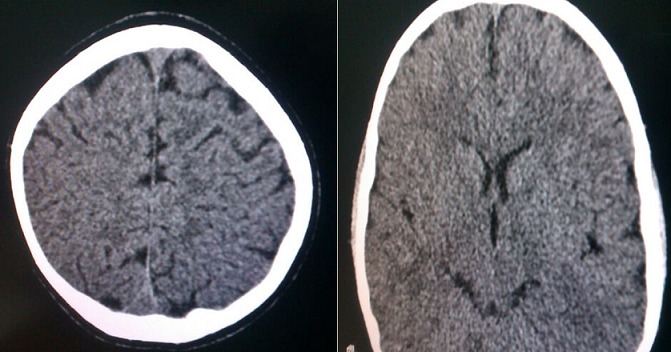
Image du CT scan montrant une résorption de l'oedème et de l'hématome

Le patient avait été soumis sous un traitement fait de Dexaméthasone (4 mg deux fois par jour pendant 3 jours puis une fois 4 mg par jour pendant 2 jours) et Piracetam (800 mg deux fois par jour pendant 14 jours). Après une semaine de traitement, l'enfant ne présentait aucune plainte et l'examen neuropsychiatrique était normal. Le scanner cérébral réalisé après deux semaines de traitement avait montré une résorption de l'oedème et de l'hématome (Figure 3). L'enfant était sorti au J18 d'hospitalisation.

## Discussion

Dans les AE par foudroiement, la littérature fait mention de l'hémorragie cérébrale, mais une telle observation a été aussi rapportée dans l’électrisation par un courant électrique à haute tension par Chaibdraa qui formula l'hypothèse étiologique selon laquelle l'AE par contact avec un câble transporteur de courant électrique à haute tension est envisagé comme étiologie de l'hémorragie cérébro-méningée sur base des éléments suivants: (a) l'exposition à un courant de haute tension, (b) la perte de connaissance brève initiale, (c) l'existence de lésions de brûlures cutanées témoignant du passage du courant par l'extrémité céphalique homolatérale à l'hémorragie cérébrale, (d) la survenue de l'hémorragie cérébrale dans le délai de trois jours suite à l'accident sans lésion osseuse, (e) l'existence réelle de cette complication chez les victimes de foudroiement, qui est un AE [9]. Dans notre observation, aucune lésion osseuse n'a été observée mais nous ne pouvons donner avec exactitude le délai d'installation de l'hémorragie cérébrale après AE du fait que le scanner cérébral à notre possession a été réalisé qu’à deux mois après l'AE. Cette hypothèse étiologique reste toujours valable dans notre observation du fait qu'aucun antécédent ou évènement pouvant à l'origine d'une hémorragie cérébrale n'a été noté chez notre patient et que, d'après Chuang, les complications artérielles hémorragiques surviennent jusqu′à six semaines après l′accident initial [10]. Cette observation confirme le mécanisme physiopathologique de survenue des manifestations neurologiques centrales en relation avec les effets de l’électricité de haute tension sur le parenchyme cérébral qu'avait décrit Chaibdraa [9]. Les hémorragies sont secondaires à la fragilisation de la paroi vasculaire et les artères de petit calibre sont plus souvent atteintes. Les vaisseaux et les nerfs offrent la moindre résistance et sont donc le siège d'une intensité la plus forte, d'où il est reconnu que le trajet de l’électrisation est préférentiellement vasculo-nerveux. L’électricité possède un rôle spécifique sur les vaisseaux, en particulier sur les artères à l'origine de thromboses, d'une fragilisation de la paroi des petites artères pouvant se rompre occasionnant des hémorragies. Ces complications artérielles hémorragiques surviennent jusqu′à six semaines après l′accident initial, alors que les anévrismes peuvent se développer des mois plus tard [3, 10].

Selon la littérature, les études cliniques les plus approfondies des manifestations neurologiques des AE sont faites à partir des accidents post-foudroiement [7, 9, 11] et Cherington [3, 12] classe les atteintes neurologiques secondaires en quatre grands groupes en fonction de la date d'apparition et du mode d’évolution. Le groupe 1 est fait des lésions immédiates et transitoires et composé de perte de connaissance brève, confusion, céphalées, amnésie, paresthésie et asthénie. La paralysie prédominante aux membres inférieurs (kéraunoparalysie de Charcot) s′accompagne de pâleur, d′une vasoconstriction sévère avec diminution des pouls et d′une hypertension artérielle. D'après l'anamnèse, ces quatre premiers signes ont été aussi notés chez notre patient. Cherington souligne que la perte de connaissance brève est le signe le plus fréquemment retrouvé [13]. Le groupe 2 comprend des lésions immédiates, prolongées et permanentes et définit des lésions cérébrales, médullaires et périphériques. Au niveau du cerveau, on note une encéphalopathie post-arrêt cardiaque, des zones d′infarcissement, un oedème cérébral, une hémorragie, une atrophie cérébelleuse, des hématomes; une myélopathie avec démyélinisation et une lésion cordonale sont notées au niveau médullaire; les déficits périphériques sont faits d'une destruction des cellules de Schwann (fragmentation axonale), un syndrome de compression nerveuse (oblitération vasculaire et fibrose périneuronale). Chez notre patient, les lésions de ce groupe qui ont été objectivées sont l'oedème cérébral et l'hémorragie cérébro-méningée mais nous avons aussi noté une atteinte cérébelleuse. Cette hémorragie survient durant les trois premiers jours à six semaines consécutifs à l'accident initial [3, 9, 10] et reste permanente pendant plusieurs jours. Le groupe 3 est composé des lésions retardées et progressives et intéresse les manifestations à long terme dont les mécanismes physiopathologiques sont méconnus. Elles peuvent survenir plusieurs mois après l′accident initial et sont progressives: neuropathie périphérique, paresthésie, parésie, atrophie optique, dysfonction cérébelleuse, épilepsie, myélite transverse, paraplégie, dépression et troubles de la mémoire et de la concentration [3, 14]. C'est ainsi que chez notre patient nous avons pu observer, deux mois après l'AE, une atteinte cérébelleuse caractérisée par une incoordination des mouvements, une marche ébrieuse associé à un trouble mnésique avec altération de la mémoire et désorientation temporo-spatiale. C'est cet aspect clinique qui constitue la particularité dans notre observation. Le groupe 4 est constitué des lésions neurologiques associées. Ce sont les pathologies liées aux traumatismes associés et peuvent se résumer en traumatisme crânien et section médullaire. Chez notre patient, aucune de ces pathologies n'avait été observée.

S'agissant de l’évolution, la morbidité engendrée par les AE est considérable et des séquelles en particulier neurologiques et psychologiques qui peuvent être très invalidantes [15]. Comme pour le patient âgé de 37 ans victime d'un AE par un courant électrique à haute tension décrit par Chaibdraa [9], nous avons aussi noté chez notre patient une bonne récupération clinique sans séquelles et les examens paracliniques de contrôle se sont révélés normaux.

## Conclusion

L'hémorragie cérébro-méningée peut être la conséquence de l'exposition à un courant de haut voltage. Cette observation confirme le mécanisme physiopathologique de survenue des manifestations neurologiques centrales en relation avec les effets de l’électricité de haute tension sur le parenchyme cérébral décrit par Chaibdraa. En plus, la symptomatologie peut être faite tardivement des manifestations neuropsychiatriques tardives et progressives, d'où un suivi médical continu doit être proposé aux patients victimes d'AE.
